# Agonistic Anti-CD40 Antibody Triggers an Acute Liver Crisis With Systemic Inflammation in Humanized Sickle Cell Disease Mice

**DOI:** 10.3389/fimmu.2021.627944

**Published:** 2021-03-04

**Authors:** Ayla Yalamanoglu, Irina L. Dubach, Nadja Schulthess, Giada Ingoglia, Delaney C. Swindle, Rok Humar, Dominik J. Schaer, Paul W. Buehler, David C. Irwin, Florence Vallelian

**Affiliations:** ^1^ Division of Internal Medicine, University of Zurich, Zurich, Switzerland; ^2^ Cardiovascular and Pulmonary Research Laboratory, Department of Medicine, University of Colorado Denver, Aurora, CO, United States; ^3^ Department of Pathology, University of Maryland School of Medicine, Baltimore, MD, United States; ^4^ Center for Blood Oxygen Transport and Hemostasis, Department of Pediatrics, University of Maryland School of Medicine, Baltimore, MD, United States

**Keywords:** sickle cell anemia, CD40, macrophage, liver disease (Ld), vasoocclusive crisis

## Abstract

Sickle cell disease (SCD) is an inherited hemolytic disorder, defined by a point mutation in the β-globin gene. Stress conditions such as infection, inflammation, dehydration, and hypoxia trigger erythrocyte sickling. Sickled red blood cells (RBCs) hemolyze more rapidly, show impaired deformability, and increased adhesive properties to the endothelium. In a proinflammatory, pro-coagulative environment with preexisting endothelial dysfunction, sickled RBCs promote vascular occlusion. Hepatobiliary involvement related to the sickling process, such as an acute sickle hepatic crisis, is observed in about 10% of acute sickle cell crisis incidents. In mice, ligation of CD40 with an agonistic antibody leads to a macrophage activation in the liver, triggering a sequence of systemic inflammation, endothelial cell activation, thrombosis, and focal ischemia. We found that anti-CD40 antibody injection in sickle cell mice induces a systemic inflammatory and hemodynamic response with accelerated hemolysis, extensive vaso-occlusion, and large ischemic infarctions in the liver mimicking an acute hepatic crisis. Administration of the tumor necrosis factor-α (TNF-α) blocker, etanercept, and the heme scavenger protein, hemopexin attenuated end-organ damage. These data collectively suggest that anti-CD40 administration offers a novel acute liver crisis model in humanized sickle mice, allowing for evaluation of therapeutic proof-of-concept.

## Introduction

Sickle cell disease (SCD) is a monogenic autosomal recessive disorder defined by a missense mutation in the β-globin gene, forming the sickle hemoglobin (HbS) (1). Affecting nearly 300’000 newborns per year with the highest prevalence in sub-Saharan Africa, India, and the Mediterranean and Middle East regions, SCD imposes a considerable global health burden (2, 3). The substitution of glutamic acid with the hydrophobic amino acid valine at position 6 in the β-globin gene causes erythrocyte hemoglobin to polymerize and facilitate red blood cells (RBCs) sickling under deoxygenation (4). Sickled RBCs demonstrate abnormally adhesive properties and impaired deformability (5). As a result, damaged erythrocytes hemolyse and release hemoglobin into the circulation promoting NO-scavenging, oxidative damage, iron overload, and organ dysfunction (6–8).

The term “sickle cell crisis” summarizes clinically heterogeneous acute disease complications such as vascular-occlusive crisis, hemolytic crisis, sequestration syndrome with enlargement of liver and spleen and, aplastic or hypoplastic crisis (1, 9). It is associated with life-threatening conditions such as acute chest syndrome (ACS), stroke, avascular necrosis, renal dysfunction, aplastic, and splenic sequestration crisis. Known inciting factors for a sickle crisis are hypoxia, dehydration, stress, and infection (10, 11). The sequelae of aggravated hemolysis, hypercoagulability and, increased adhesion of RBCs, leukocytes, and platelets to the endothelium aggravate local hypoxia and result in vaso-occlusive crisis (VOC) and end-organ ischemia (1, 7, 12, 13).

Humanized sickle mice have been developed for preclinical studies of SCD. The Berkeley mouse model has targeted deletions of murine α and β globins that are compensated by a transgene containing human α, γ, and β^S^ globin (14). Phenotype similarities to human SCD are erythrocyte sickling, extravascular and intravascular hemolysis, severe anemia, and multiorgan infarcts mainly reported in liver, kidney, and spleen (14, 15). Hypoxia-reoxygenation, systemic administration of lipopolysaccharides (LPS) or tumor necrosis factor-α (TNF-α) have been used in preceding studies to induce acute vaso-occlusion and sickle crisis in murine models of SCD (13, 16–21). These models allow for the study of vaso-occlusion within the microcirculation; however, all these models have significant limitations such as high variability, animal welfare considerations (e.g., dehydration-triggered crisis), or poorly defined pathophysiological pathways.

CD40 belongs to the tumor necrosis factor receptor (TNF-R) superfamily and is mainly expressed on B-cells and antigen-presenting cells (22) (23). The CD40 transduced signal activates the canonical NFkB-pathway and comprises a key pathway of immune cell communication (24). Soluble CD40 ligand (sCD40L) is elevated in SCD and increases during a crisis and in patients with acute chest syndrome (25–27). The CD40-CD40L pathway may contribute to the chronic inflammatory state of SCD as well as to the initiation and propagation of sickle crisis. In this study, we found that activation of CD40 signaling by agonistic anti-CD40 antibody in Berkeley SCD mice leads to an acute and phenotypically distinct disease state with systemic inflammation, severe vaso-occlusive liver disease, and right heart dysfunction. As a validation of our model, we found that the treatment of SCD mice with the TNF-α blocker etanercept or plasma-derived human hemopexin significantly reduced anti-CD40-induced acute inflammation and liver disease.

## Methods

### Animal Model

Mice 12 to 16 weeks of age were used. The Berkeley sickle mice with genotype (Tg(Hu-miniLCR α1GγAγδβS) Hba^0/0^, Hbb^0/0^, and hemizygous for Tg(HBA-HBBs)41Paz (non-sickling mice from colony) were obtained from the Jackson Laboratory. All mice were housed and bred under specific-pathogen-free conditions in the Laboratory Animal Services Center (LASC) of the University of Zurich.

All experimental protocols were reviewed and approved by the Veterinary Office of the canton of Zurich. All animals were maintained at the animal facility of the University of Zurich (LSC) and were treated in accordance with guidelines of the Swiss federal Veterinary Office.

### Mouse Treatments

#### Mouse Anti-CD40 Antibody Treatment

Mice were treated intraperitoneally (i.p.) with 20 mg/kg of an agonistic anti-CD40 antibody (InVivoMab, clone FGK4.5/FGK45 BioXCell). 30 h after anti-CD40 antibody injection, blood was removed by terminal heart puncture or blood withdrawal from the vena cava inferior and liver tissue was collected for histology or stored at - 80°C until further use.

#### Hemopexin and Etanercept Treatment

Mice were treated subcutaneously (s.c.) with 3 mg human plasma-derived hemopexin (CSL Behring, batch TO342022B, 92 mg/ml) 5 days a week, for 3 weeks (a total of 17 injections) before anti-CD40 treatment. Our studies (unpublished data) have evaluated hemopexin at 50 mg/kg, 100 mg/kg and 300 mg/kg administered subcutaneously, three times per week. These studies were conducted in Berkeley SCD mice with progressive cardiopulmonary dysfunction. A dosing strategy of 300 mg/kg hemopexin, subcutaneous, three times per week was the most effective regimen to maintain a mean +/- SD steady state plasma concentration of 3.30 +/- 0.85 mg/ml and correct disease progression. To neutralize TNF-α, etanercept (Enbrel, 25mg/0.5ml, Pfizer PAA044617, Lot W47929) 100mg/kg was injected intraperitoneal (i.p.) on day 1, and 3 followed by anti-CD40 antibody 2 h after etanercept administration on day 3. Mice were harvested 30 h after anti-CD40 injections. The dose of etanercept used in this study is based on human equivalent dosing recommendations.

### Hepatic Enzyme Analysis

Alanine aminotransferase (ALT) and Bilirubin levels (Reflotron; Roche) were measured from mice plasma after anti-CD40 antibody treatment. Plasma lactate dehydrogenase (LDH) measurements were performed by the Veterinary Laboratory of the University of Zurich.

### Bio-Plex Cytokine Assays

Concentrations of cytokines and chemokines IL-6, IL-12p40, CCL2 (MCP-1), CCL3 (MIP-1α), CCL4 (MIP-1β), TNF-α were determined with Bio-Plex Cytokine Assays (Bio-Rad). sE-selectin, ICAM, PAI-1, sP-selectin, and proMMP9 were determined using Milliplex Map mouse cardiovascular disease (CVD) Magnetic Bead Panel 1, Merck Millipore.

The assay was analyzed with a Bio-Plex 200 system (Bio-Rad). The results were analyzed using Bio-Plex Data Pro software (Bio-Rad).

### RT-qPCR

Real-time PCR was performed according to a standard workflow on a 7500 Fast Real-Time PCR System (Applied Biosystems).

#### Primer Sequences (5’-3’)

Hprt1 forward: cctcctcagaccgcttttt, reverse: aacctggttcatcatcgctaa

Cd40 forward: aaggaacgagtcagactaatgtca, reverse:agaaacaccccgaaaatggt

Il6 forward: gctaccaaactggatataatcagga, revever: ccaggtagctatggtactccagaa

Il12b forward: agttgacggaccccaaaag, reverse: agctggatgctctcatcagg

Ccl2 forward:catccacgtgttggctca, reverse:gatcatcttgctggtgaatgagt

Cxcl9 forward: cttttcctcttgggcatcat, reverse: gcatcgtgcattccttatca

Cxcl10 forward: gctgccgtcattttctgc, reverse: tctcactggcccgtcatc

### Tissue Iron Measurements

Kidney samples cut to a weight of 30**–**35 g were homogenized in double deionized H2O at 1:10 wt/vol. Homogenates were mixed with 500 μl of an acid mixture containing 1mM HCl and 10% Trichloroacetic acid (TCA), and incubated at 50°C for 1 h with intermittent shaking (28). The samples were then centrifuged at 15,000 × g for 15 min at room temperature. The clear supernatant (90 μl) was mixed with 30 μl of 20 mg/ml ascorbic acid followed by 20 μl of ferrozine (0.85% wt/vol in hydroxylamine hydrochloride) (29). The samples were allowed to completely develop for 30 min. The absorbance was measured at 562 nm using the plate reader infinite M200 Pro Tecan. A standard curve was generated using an iron standard (500 μg/dl).

### Cardiac Measurements

At the conclusion of the study, mice underwent terminal open chest right ventricular (RV) function measurements with a 1.2F, FTE-1212B-4018 pressure volume catheter (Transonic Systems Inc., Ithaca, NY) inserted by direct cardiac puncture. Mice were induced inhaled isoflurane (4%**–**5%), and tracheal incision (~ 1 cm) was performed. Next, a tracheal tube was inserted and connected to an Anesthesia Workstation or Hallowell EMC Microvent and an anesthetic plain was maintained at 1.0%**–**2.5% isoflurane in 100% oxygen. After which, a thoracotomy was performed exposing the heart, the pericardium was resected and a small hole made at the base of the RV with a 30g needle for insertion of the pressure-volume catheter. Steady state hemodynamics are collected with short pauses in ventilation (up to 10 s) or high frequency oscillatory ventilation to eliminate ventilator artifacts from the pressure-volume recordings. Occlusions of the inferior vena cava were performed by applying pressure to the inferior vena cava (up to 10 s) through the abdominal opening. After pressure volume and hemodynamic measurements were completed, mice were humanely euthanized by exsanguination and cervical dislocation. Data was recorded continuously using LabScribe2 and analyzed offline.

### Nonparenchymal Liver Cell Isolation

Liver digestion was performed with a protocol modified from Cabral et al. (30, 31). The abdominal cavity of a living, deeply anesthetized mouse was opened, and the portal vein was catheterized for *in situ* liver perfusion and digestion with collagenase B buffered solution (Roche, 11088815001). The livers were dissected and the mouse sacrificed. The digested liver was mechanically disaggregated in a petri dish on ice and filtered through a 100 μm cell strainer. The cell suspension was centrifuged twice at 60 x g for 2 min at 4°C, and the pellets of hepatocytes were discarded. The supernatant was then centrifuged at 300 x g for 5 min at 4°C to obtain a pellet of nonparenchymal liver cells, containing endothelial cells. Nonparenchymal liver suspensions were filtered through a 70-μm cell strainer (Sigma Aldrich, cat. n. CLS431751) and centrifuged at 300 x g for 5 min and resuspended in the FACS buffer.

### Flow Cytometry Sample Preparation and Analysis

Flow cytometry has been performed according to standard protocols (32). Stained cells were analyzed using an LSRFortessa (BD). Data was analyzed using FlowJo software.

Antibodies: Endothelial cells: Pacific Blue anti-CD45 (0.5 mg/ml, BioLegend 109820), FITC anti-CD102 (ICAM, 0.5 mg/ml BD Pharmingen, 557444), APC anti-CD31 (PECAM, 0.2 mg/ml, BD Pharmingen 551262), PE anti-VCAM1 (0.2 mg/ml, BioLegend 105714).

### Histology

An Olympus IX71 microscope was used for macroscopic photographs of fresh livers. Kidney and liver were fixed in 10% formalin for 24 h and stored in 100% isopropanol. Tissue was embedded in paraffin, and 2- to 5-μm sections were prepared. Liver sections were stained with standard hematoxylin-eosin (H/E) procedures.

### Nonheme Iron Histopathology

Kidney sections were incubated with Perls iron reagent containing 5% potassium ferrocyanide and 2% hydrochloric acid for 45 min at room temperature and rinsed in deionized water. Sections were then incubated with 0.3% hydrogen peroxide and 0.01 M sodium azide in methanol for 30 min at room temperature. All sections were then rinsed in 0.1 M PB, pH 7.4, washed in deionized water, and lightly counterstained with Gill’s II hematoxylin (33). The stained sections were imaged using a Zeiss Apotome.2 microscope.

### Statistical Analysis

Data plotting and statistical analysis were performed with Prism 8 (GraphPad). Two-way hierarchical clustering analyses were performed with the Ward algorithm as provided by JMP14 software (SAS). UMAP analysis was performed using BioVinci (2.8.5). The following parameters were applied: decision tree: Gini, cost complexity pruning: 0.04, number of neighbors: 15, metric: euclidean, minimum distance between embedded points: 0.1. For group comparisons of other data types, we used ANOVA with Tukey’s post test as indicated in the figure legends. All data points are displayed in the graphs.

*p < 0.05, **p < 0.01, ***p < 0.001, ****p < 0.0001.

## Results

### Agonistic Anti-CD40 Antibody Triggers Acute Vaso-Occlusive Hepatitis in Sickle Cell Mice

**Figure 1:**
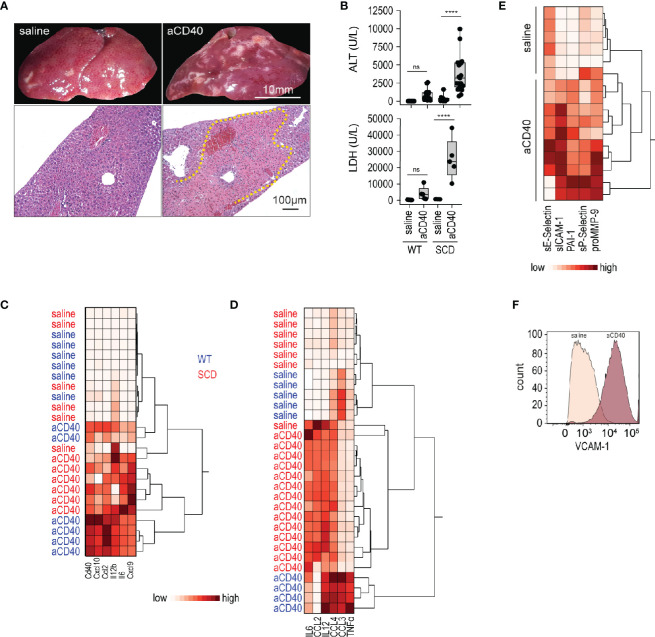
Vaso-occlusive hepatitis and systemic inflammation after anti-CD40 antibody treatment in sickle cell mice. **(A)** Photographs of representative liver lobes (top panel) and histology image for H&E staining after treatment of sickle cell mouse with saline or anti-CD40 antibody. Images are taken 30 h after anti-CD40 antibody administration. Scale bar 10 mm and 100 µm, respectively. Dotted yellow line marks extended infarct area. **(B)** Plasma alanine transaminase (ALT) and lactate dehydrogenase (LDH) concentrations in heterozygote (WT) and homozygote (SCD) Berkeley mice treated with saline or anti-CD40 (aCD40) antibody (groups; for ALT WT saline n=12, WT aCD40 n=16, SCD saline n=12, SCD aCD40 n=19, for LDH n=5). **(C)** Hierarchical clustering analysis of the relative mRNA expression of key inflammatory genes measured by RT-qPCR in whole liver of WT (blue) or SCD (red) mice treated or not with anti-CD40 antibody (white=low expression, red=high expression) (n=6). **(D)** Hierarchical clustering analysis of plasma cytokines from saline and anti-CD40 antibody treated WT (blue) or SCD (red) mice (white=low expression, red=high expression) (groups; saline n=5–7, aCD40 n=4–14). **(E)** Hierarchical clustering analysis of plasma soluble adhesion molecules, plasminogen activator inhibitor 1 (PAI1), and matrix metalloproteinase-9 (pro MMP9) from saline and anti-CD40 treated sickle cell mice (white=low expression, red=high expression) (groups; saline n=6, aCD40 n=10). **(F)** Flow cytometry histogram of nonparenchymal liver cell suspensions gated for VCAM-1 in saline or anti-CD40 treated sickle cell mice. The displayed cells were gated from live CD45^-^, CD31^+^, and CD102^+^ cells. Data are representative of three independent experiments. Each data point represents a single mouse. ****p < 0.0001 for all panels. All comparisons between control (saline) and anti-CD40 dosed sickle cell mice were conducted by a two-tailed student’s t -test with significance set at a significance of p < 0.05.

We treated SCD (Hbb-/-) and wild-type (Hbb +/-) SCD mice with a single intraperitoneal (i.p.) injection of an agonistic anti-CD40 antibody. 30 h after injection, we observed extended areas of white spots on the liver surface. These areas were indicative of large necrosic zones with occlusive fibrin thrombi observed by light microscopy of H&E stained liver sections (yellow dotted line, **Figure 1A**). This liver pathology was accompanied by a significant increase of liver enzymes and lactate dehydrogenase (LDH) levels in the plasma, demonstrating an expected pathophysiological response in this model (**Figure 1B**).

Gene expression of pro-inflammatory cytokines and myeloid activating markers were markedly induced in the whole liver RNA of anti-CD40-dosed SCD and WT mice (**Figure 1C**), suggesting a strong immune activation.

### Anti-CD40 Injection Aggravates Systemic Inflammation and Endothelial Activation in Sickle Cell Mice

To determine systemic inflammatory changes after anti-CD40 antibody treatment, we measured pro-inflammatory cytokines/chemokines in plasma. As reflected by the hierarchical clustering analyses in **Figure 1D**, we observed clear segregation of saline and antibody-treated animals, which was indicative of a systemic inflammatory response in the anti-CD40 dosed SCD and WT mice.

Endothelial cell activation is a hallmark of SCD pathology (34) that precipitates the vaso-occlusive process. Endothelial cell activation markers increased significantly in the plasma of anti-CD40 dosed SCD mice compared to saline-treated animals (**Figure 1E**). Accordingly, flow cytometry of liver cell suspensions revealed an upregulation of vascular cell adhesion molecule (VCAM-1) on endothelial cells, confirming that anti-CD40-treatment ultimately leads to an endothelial cell activation (**Figure 1F**).

### Anti-CD40 Antibody Induces Hyperhemolysis in Sickle Cell Mice

**Figure 2:**
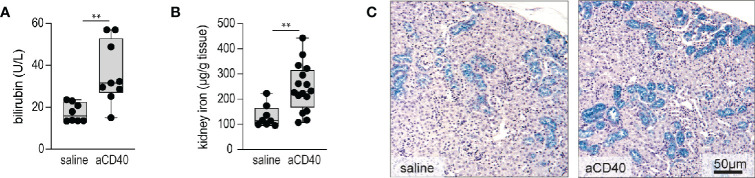
Accelerated hemolysis after anti-CD40 administration in sickle cell mice. **(A)** Plasma bilirubin in saline versus anti-CD40 treated sickle cell mice (groups; saline n=8, aCD40 n=9). **(B)** Iron concentration measured by the ferrozine method in kidneys of saline versus anti-CD40 treated sickle cell mice (groups; saline n=8, aCD40 n=16). **(C)** Representative histology images for Perls staining of kidney sections from saline and anti-CD40 treated sickle cell mice. Blue color represents non-heme iron deposits, mainly seen in proximal tubules. Scale bar 50 µm. Each data point represents a single mouse. ** *p*<0.01, * *p*<0.05, all comparisons between control (saline) and anti-CD40 dosed sickle cell mice were conducted by a two-tailed student’s t -test with significance set at a significance of p<0.05.

Accelerated hemolysis with hemoglobinuria is a key feature of sickle crisis (6). Quantification of hemolysis in SCD is confounded by RBC lysis during blood sampling. Therefore, we have estimated the hemolytic activity by plasma bilirubin and renal iron content, assuming that acute changes in renal iron directly reflect hemoglobinuria and thus intravascular hemolysis. Furthermore, we stained kidney sections with Perls reagent to visualize the distribution of iron (35). Bilirubin levels and kidney iron significantly increased following anti-CD40 dosing (**Figures 2A–2B**). Perls staining revealed increased iron deposition in the proximal tubules of anti-CD40 dosed SCD mice compared to the vehicle dosed animals (**Figure 2C**).

### Right Ventricular Dysfunction and Right Heart Failure After Anti-CD40 Administration in Sickle Cell Mice

**Figure 3:**
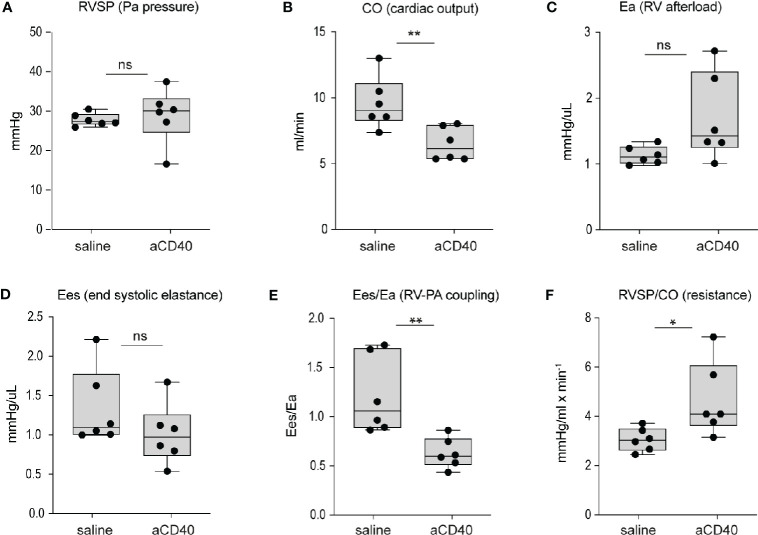
Hemodynamic changes after anti-CD40 administration in sickle cell mice. Cardiovascular parameters in sickle cell mice following saline (control) and anti-CD40 antibody dosing. **(A)** RSVP - pulmonary arterial pressure (p=0.723, n=6). **(B)** CO - Cardiac Output (p= 0.0089**, n=6). **(C)** Ea - Right ventricular (RV) afterload (p=0.0652, n=6). **(D)** Ees - end systolic elastance (p=0.2257, n=6). **(E)** Ees/Ea - RV to pulmonary artery (PA) coupling (RV-PA) (p=0.0069**, n=6). **(F)** RVSP/CO - RV systolic pressure (SP) divided CO (p=0.0312*, n=6). Right ventricular wall stiffness was also significantly greater (p=0.039*, n=6) in anti-CD40 compared to control sickle cell mice (data not shown). **p < 0.01, *p < 0.05, all comparisons between control (saline) and anti-CD40 dosed SCD mice were conducted by a two-tailed student’s t -test with significance set at a significance of p<0.05.

Aggravated hemolytic anemia, vaso-occlusion in the liver and systemic inflammation occur after anti-CD40 dosing, prompting us to consider cardiac changes associated with these effects. We evaluated cardiac abnormalities using solid state catheter pressure-volume measurements (PV loops). We measured PV loops of the right ventricle (RV) 30 h after injection with either saline or anti-CD40 antibody. We did not observe significant changes in either right ventricular systolic pressures (RVSP), contractility (Ees), or RV afterload (Ea). However, we noted a 35% decrease in cardiac output and a corresponding rise in pulmonary vascular resistance, suggesting anti-CD40 alters pulmonary vascular function acutely. The RV to pulmonary artery vascular coupling ratio determined by the fraction obtained from Ees/Ea describes the efficiency of energy transfer between the RV and PA. Under normal conditions this value remains between 1 and 2 (36). In the present study SCD mice dosed with anti-CD40 demonstrated a suppressed Ees/Ea of approximately 0.53 suggesting an acutely uncoupled and inefficient RV (**Figures 3A–3F**).

### Effects of TNF-α Blockade and Heme Scavenging Following Anti-CD40 Exposure in Sickle Cell Mice

**Figure 4:**
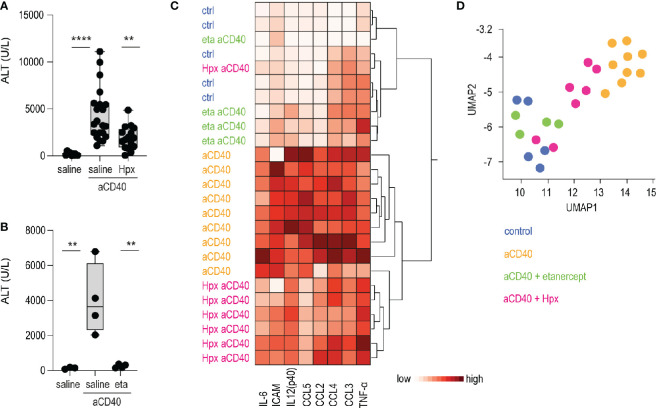
Hemopexin and etanercept treatments as validation of the model. **(A)** Plasma ALT concentrations from saline and plasma-derived hemopexin (Hpx) treated sickle cell mice dosed or not with anti-CD40 antibody (groups; saline n=12, aCD40 n= 21, hpx aCD40 n=16). **(B)** Plasma ALT concentrations from saline and etanercept (eta) treated sickle cell mice dosed or not with anti-CD40 antibody (n=4). **(C)** Hierarchical clustering analysis of plasma cytokines from saline, hemopexin (Hpx), etanercept (eta) dosed sickle cell mice, treated or not with anti-CD40 antibody (white=low expression, red=high expression) (groups; saline n=5, aCD40 n=9, hpx aCD40 = 7, eta aCD40 n=4). **(D)** UMAP plot showing color-coded plasma cytokines from the same groups of animals as in **(C)** ****p < 0.0001, **p < 0.01 ANOVA with Tukey’s *post hoc* test for **(A, B)**.

Etanercerpt blocks the binding of TNF-α to the TNF receptor, inhibiting its proinflammatory signaling (37). Hemopexin is the primary heme scavenger protein in plasma, which irreversibly binds and inactivates oxidative and proinflammatory activities of cell-free heme (38, 39). Both molecules have shown to mitigate inflammation and vaso-occlusion in SCD mice (40–43). To validate our anti-CD40 antibody-induced vaso-occlusive liver disease as a novel crisis model, we pretreated animals with etanercept or plasma-derived hemopexin. The TNF-α blocker was injected i.p. on day 1 and 3, followed on day 3 by anti-CD40 treatment. Hemopexin was administered over 3 weeks subcutaneously (sc) 5 days a week prior to anti-CD40 administration to achieve steady-state plasma concentration leading to an extended attenuation of heme-toxicity. In parallel, we evaluated vehicle injected SCD mice as experimental controls. To assess disease activity, transaminase levels and proinflammatory biomarkers were measured in plasma. After anti-CD40 dosing, ALT concentrations were significantly attenuated in both hemopexin or etanercept pretreated animals compared to vehicle controls (**Figures 4A, B**). These findings indicate that TNF-α blockade and heme scavenging attenuate the susceptibility to an anti-CD40 induced acute liver crisis.

Measurement of proinflammatory cytokines, chemokines and macrophage-activating markers in plasma were broadly suppressed in etanercept pretreated animals. This suppressive effect on individual inflammatory markers was less pronounced in SCD mice that received hemopexin prior to anti-CD40 dosing. However, the hierarchical clustering and UMAP analysis shown in **Figures 4C, D** clearly segregated the hemopexin treated animals in a distinct group with a less pronounced inflammatory plasma signature.

## Discussion

The present study defines a novel murine model of inflammation-induced hepatic crisis in sickle cell mice. In response to agonistic anti-CD40 antibody treatment, sickle cell mice developed an acute hepatitis with histological features of vessel-occlusion and ischemia. The liver pathology was accompanied by systemic inflammation, accelerated hemolysis, and acute right heart dysfunction, recapitulating cardinal characteristics of a sickle crisis (44). As further validation of our model, we found that the treatment of sickle cell mice with the TNF-α blocker etanercept or plasma-derived human hemopexin significantly reduced anti-CD40-induced acute inflammation and liver necrosis. Collectively, our observations suggest that anti-CD40 exposure induces an acute liver crisis in sickle cell mice and that this model may be valuable to evaluate potential therapeutics.

A defining feature of the present model is characterized by vaso-occlusive necro-hepatitis leading to a massive increase in liver enzymes mimicking acute hepatic crisis, occurring in approximately 10% of patients with SCD (45). In wild-type mice, treatment with an agonistic anti-CD40 antibody triggers a hyperinflammatory syndrome with a cytokine storm and limited liver toxicity (46). We have previously demonstrated that CD40 ligation on liver macrophages initiates a pathophysiological cascade of endothelial cell activation, disseminated intravascular coagulation (DIC), vaso-occlusion, resulting in liver ischemia. In wild-type mice, this sequence was abolished in mice with a lineage-selective deletion of CD40 in macrophages.

Anti-CD40 antibody treatment induced a systemic inflammatory response in wildtype and SCD mice. Plasma cytokines suggested a different pattern and possibly less pronounced systemic inflammation in sickle cell mice compared to wild-type littermates after anti-CD40 treatment. This finding is consistent with the previous observation that liver macrophages in hemolytic mice are skewed toward a hypoinflammatory phenotype (30, 47). Nevertheless, CD40-induced inflammation led to an exaggerated systemic response with endothelial cell activation and hemolysis culminating in an acute liver crisis in the SCD mice. Triggering toll-like receptor (TLR4), heme is an established propagator of vaso-occlusion and the acute chest syndrome in mouse models of sickle cell disease (43, 48, 49). Hemopexin irreversibly sequesters heme in a hexa-coordinated protein complex aborting oxidative and proinflammatory activities of hemoglobin and heme (38, 39, 42, 43, 50). The positive effect of hemopexin treatment in our studies reinforces the role of accelerated hemolysis and heme as a disease amplifier in the CD40-induced liver crisis.

In a hepatic crisis, hypervolemia and congestive heart failure occur in response to rapid and repeated changes of liver sinusoidal pressure (51). Therefore, the adverse hemodynamic response observed in our model may be a direct result of the significant liver congestion induced by the events mentioned above.

One of the limitations of existing murine sickle cell crisis models is the lack of a simple readout to quantify disease activity. In murine SCD, hypoxia-reoxygenation models are typically associated with severe crises characterized in part by hepatopathy (52), appearing progressively after a prolonged period of hypoxia and graded by a multifactorial scoring system. Other approaches to crisis induction in murine SCD include LPS and TNF-α treatments, whose evaluation of disease is mainly based on invasive methods such as intravital microscopy techniques to quantify blood flow stasis in the skin vasculature (13). In our model, we found an excellent correlation between ALT level and disease extent, providing a simple and accurate quantitative readout for the model supporting the preclinical screening of new drugs.

Transgenic sickle cell mice are fragile and prone to morbidity and mortality making any mechanistic study challenging. Unlike LPS and TNF-α, anti-CD40 antibody has a long and predictable half-life *in vivo* (53). Moreover, CD40 expression is more restricted than the expression of toll-like receptors or the TNF-α receptor. Both elements appear to be critical contributors to the high reproducibility of the anti-CD40-induced inflammatory response.

In humans, sickle crisis ranges in duration from several hours up to days. While individual crises themselves typically resolve with supportive care and close monitoring, repetitive crises are a major contributor toward progessive disease and significant morbidity. Therefore, studying therapeutic approaches to limit crises induction in rationally designed and translatable animal models is critical to effectively attenuating SCD sequela progression in humans.

In summary, this novel anti-CD40-triggered liver crisis model provides an additional pre-clinical option to improve the understanding of sickle cell crisis physiopathology. Further, this model offers an efficient approach toward supporting proof-of-concept studies that evaluate therapeutic approaches to limit the duration of SCD crisis.

## Data Availability Statement

The original contributions presented in the study are included in the article/supplementary material. Further inquiries can be directed to the corresponding author.

## Ethics Statement

The animal study was reviewed and approved by cantonal veterinary office.

## Author Contributions

AY performed the experiments, analyzed the data, and wrote the paper. ID performed the experiments. NS performed the experiments. GI performed the experiments. DCS performed the experiments. RH analyzed the data and wrote the paper. DJS analyzed the data and wrote the paper. PB performed the experiments, analyzed the data, and wrote the paper. DI performed the experiments, analyzed the data, and wrote the paper. FV designed the study, performed the experiments, analyzed the data, and wrote the paper. All authors contributed to the article and approved the submitted version.

## Funding

This study was supported by Innosuisse 19300.1 PFLS-LS - 1 (to DJS), NIH/NHLBI - R01 HL125642-01 (to DCI), by the Promedica Foundation (to FV), the Olga Mayenfisch Stiftung (to FV), and the Novartis Foundation (to FV).

## Conflict of Interest

The authors declare that the research was conducted in the absence of any commercial or financial relationships that could be construed as a potential conflict of interest.
